# Forecasting climate change impacts on neotropical *Myotis*: Insights from ecological niche models for conservation strategies

**DOI:** 10.1002/ece3.11419

**Published:** 2024-06-25

**Authors:** Karoliny de Oliveira, Roberto Leonan M. Novaes, Marcelo M. Weber, Ricardo Moratelli

**Affiliations:** ^1^ Fundação Oswaldo Cruz Fiocruz Mata Atlântica Rio de Janeiro Brazil; ^2^ Programa de Pós‐Graduação Em Biodiversidade e Biologia Evolutiva Universidade Federal do Rio de Janeiro Rio de Janeiro Brazil; ^3^ Departamento de Zootecnia e Ciências Biológicas Universidade Federal de Santa Maria Rio Grande do Sul Brazil

**Keywords:** biogeography, climate change, conservation, ecological niche modeling, neotropical bats, neotropical *Myotis*, potential climate stability

## Abstract

*Myotis* originated during the Oligocene in Eurasia and has become one of the most diverse bat genera, with over 140 species. In the case of neotropical *Myotis*, there is a high degree of phenotypic conservatism. This means that the taxonomic and geographic limits of several species are not well understood, which constrains detailed studies on their ecology and evolution and how to effectively protect these species. Similar to other organisms, bats may respond to climate change by moving to different areas, adapting to new conditions, or going extinct. Ecological niche models have become established as an efficient and widely used method for interpolating (and sometimes extrapolating) species' distributions and offer an effective tool for identifying species conservation requirements and forecasting how global environmental changes may affect species distribution. How species respond to climate change is a key point for understanding their vulnerability and designing effective conservation strategies in the future. Thus, here, we assessed the impacts of climate change on the past and future distributions of two phylogenetically related species, *Myotis ruber* and *Myotis keaysi*. The results showed that the species are influenced by changes in temperature, and for *M. ruber*, precipitation also becomes important. Furthermore, *M. ruber* appears to have been more flexible to decreases in temperature that occurred in the past, which allowed it to expand its areas of environmental suitability, unlike *M. keaysi*, which decreased and concentrated these areas. However, despite a drastic decrease in the spatial area of environmental suitability of these species in the future, there are areas of potential climate stability that have been maintained since the Pleistocene, indicating where conservation efforts need to be concentrated in the future.

## Graphical abstract

This study examines the impact of climate change on *Myotis* bat species, particularly *Myotis ruber* and *Myotis keaysi*. It reveals that temperature changes significantly influence their distributions, with precipitation also playing a role for *M. ruber*. *M. ruber* shows greater adaptability to temperature fluctuations, expanding its suitable habitats, while *M. keaysi* faces contraction. Despite future challenges, areas of climate stability since the Pleistocene suggest targeted conservation efforts can be focused in specific areas.

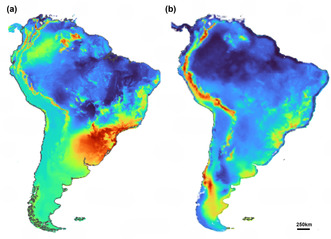

## 1 INTRODUCTION

Bats comprise one of the most successful extant mammalian orders, and *Myotis* Kaup, 1829, is the richest genera, with more than 140 species, of which ca. 35 are recognized for the Neotropics (Brown et al., 2019; Carrión‐Bonilla & Cook, 2020; Moratelli, Burgin, et al., 2019; Moratelli, Novaes, et al., 2019; Novaes et al., 2022). *Myotis* originated during the Oligocene in Eurasia underwent diversification events and dispersed across all continents (Ruedi et al., 2013). The colonization of the New World happened during the Miocene with the crossing of the Bering Strait, which during that period was a land bridge connecting Siberia to Alaska (Stadelmann et al., 2007). The fossil record of *Myotis* in South America is limited, with some of them in Argentina (Sauthier et al., 2013). The oldest fossil record dates to the Late Pleistocene, and it shows a divergence of at least 7 million years between the molecular and fossil evidence, which can be explained as a sampling bias aggravated by gaps in fossil knowledge on the continent or by preservation bias, as the diagenesis of bat fossils is hampered by the characteristics of the biology and morphology of the group (Brown et al., 2019).

*Myotis* occurs in all biogeographic regions, pointing to a high level of habitat generalism for the genus, from coniferous forests to rainforests, arid woodlands, and desert scrubs (Nowak, 1994; Weber et al., 2019). The New World *Myotis* is monophyletic and divided into two clades with a strong biogeographic association, one Nearctic and the other predominantly Neotropical (Stadelmann et al., 2007). They are divided into four species groups—lucifugus, vivesi, albescens, and ruber—showing strong cohesion between morphological and molecular data (Moratelli et al., 2013; Moratelli, Burgin, et al., 2019). However, neotropical *Myotis* has a high degree of phenotypic conservatism, especially among phylogenetically related species (Menu, 1987; Novaes et al., 2021). Therefore, the taxonomic and distributional limits of several species are poorly understood, hampering evolutionary and ecological knowledge and their application to species conservation (Novaes et al., 2023).

**FIGURE 1:**
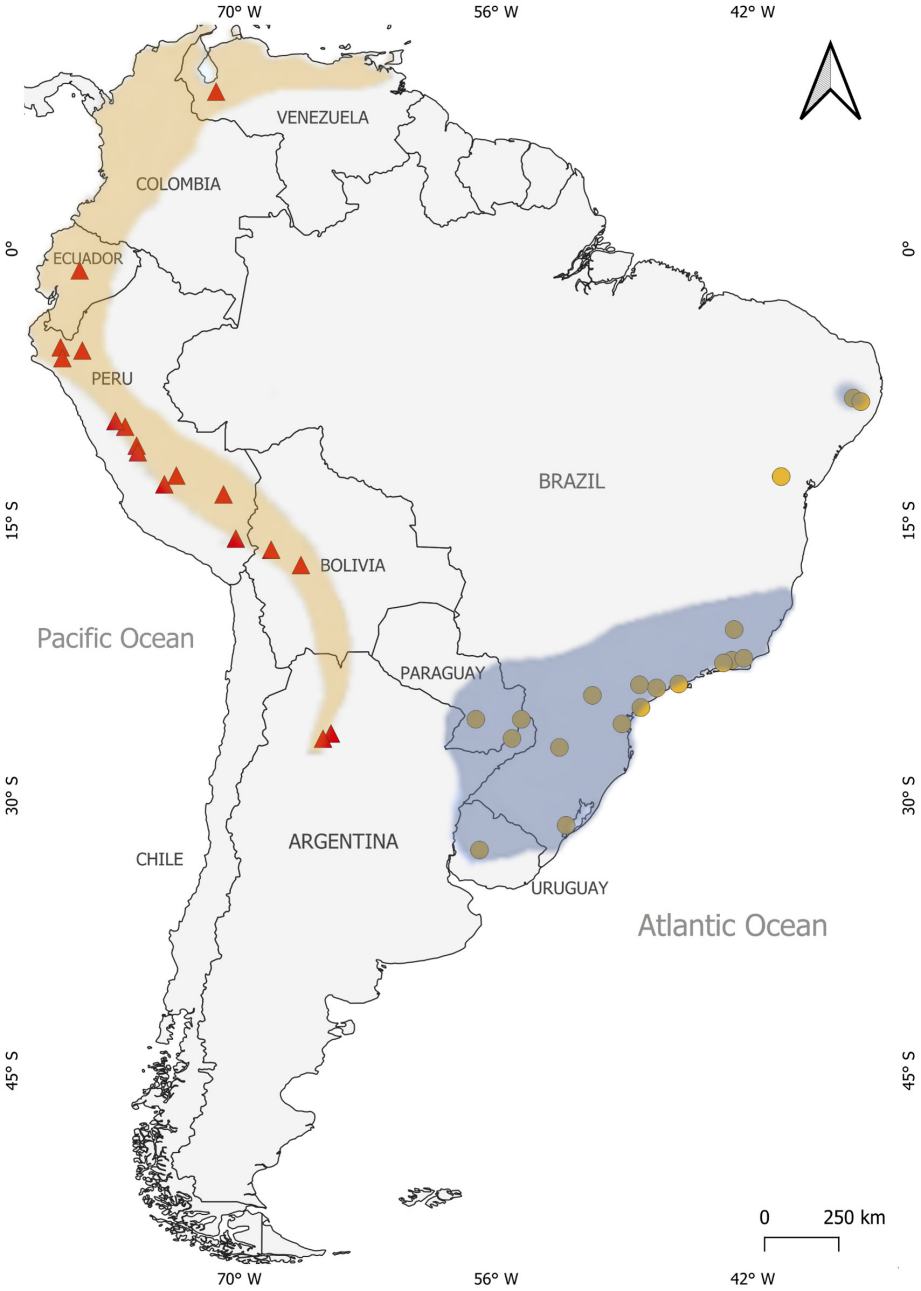
Points of occurrence of *Myotis ruber* (yellow circles) and *Myotis keaysi* (red triangles) used for ecological niche modeling. The stains represent their respective current distributions (https://www.iucnredlist.org/).

Here, we evaluate the impacts of climate change on the past and future potential distribution of two phylogenetically and phenotypically closer species, *Myotis ruber* (É. Geoffroy, 1806) and *Myotis keaysi* J.A. Allen, 1914. These two well‐defined species are restricted to South America, occurring in different environments but in parapatry in the southernmost part of their distributions (Figure 1), forming a U‐shaped continuous distribution (Moratelli et al., 2013; Moratelli, Burgin, et al., 2019). *Myotis ruber* occurs in evergreen and semi‐deciduous forests of the Atlantic Forest, from northeastern Brazil southward to northern Argentina and Paraguay, and its occurrence seems to be strongly associated with humid environments and dense forest cover (Moratelli, Burgin, et al., 2019; Weber et al., 2010). However, evidence obtained by potential distribution models based on habitat suitability suggests that these populations in northeastern Brazil may be disconnected from populations in the southern Atlantic Forest due to the absence of dispersal routes (Weber et al., 2010). *Myotis keaysi* is strongly associated with highland forests along the Andes, occurring in habitats with dense rainforest at lower elevations and seasonal forests dominated by shrubby vegetation with sparse patches of arboreal vegetation at higher elevations (Moratelli, Burgin, et al., 2019; Novaes et al., 2021). Although this species has been the subject of recent systematic revisions, its species limits and distribution are still uncertain (Carrión‐Bonilla & Cook, 2020; LaVal, 1973; Moratelli, Burgin, et al., 2019; Novaes et al., 2021).

South American ecosystems have been under different pressures for centuries, and Amazon deforestation and land use change in the Atlantic Forest from southern to Northeastern Brazil are examples of anthropogenic environmental degradation. The future of biome distribution in tropical South America in face of the synergistic combination of land use (deforestation, forest fires, and fragmentation) and climate changes, resulting in warmer and drier climates, includes savannization in portions of Amazon Forest and aridization in parts of Northeastern Brazil (Salazar et al., 2007). Furthermore, South American forest ecosystems are intrinsically more vulnerable to climate change than other ecosystems, and ongoing climate change can accelerate the loss of ecosystem resilience by promoting forest biodiversity loss and leading to another stable state with a lower density of vegetation cover (Anjos & Toledo, 2018).

Bats are a species‐rich, globally distributed group of organisms that are thought to be particularly sensitive to the effects of climate change because of their high surface‐to‐volume ratios and low reproductive rates (Festa et al., 2023). Aguiar et al. (2016), in a study with bats from the Brazilian Cerrado, evaluated the impacts of potential climate changes on the species by 2050 and concluded that if bat species were not able to move to new suitable areas and were unable to adapt, then 36 species (31.6%) could lose more than 80% of their current distribution area, and 5 species would lose more than 98% of their distribution areas in the Brazilian Cerrado. In a global study, Festa et al. (2023) showed that bats responded in varied ways, including both positive (e.g., range expansion and population increase) and negative (range contraction and population decrease), although responses to extreme events were always negative or neutral. Thus, bats represent an excellent study system for assessing the effects of climate change on vertebrates, which allows us to understand that climate change plays a more important role not only in dominating species' spatial distribution patterns but also in altering species' adaptive behaviors and survival conditions (Liu et al., 2022). In this way, identifying priority areas for conservation and habitat types preferred by the species becomes fundamental to understanding the relationship of the species with its environment and how climate changes can affect distributions (Miller, 2010; Pearson & Dawson, 2003; Weber et al., 2019).

Ecological niche modeling (ENM) has become a fundamental tool for studies that aim to recreate the relationships between species and the environments where they occur and allow us to identify unexplored areas in geography where these species might be, either from an evolutionary historical perspective or with future predictions for species conservation (e.g., Jarvie & Svenning, 2018; Leite et al., 2016; Valencia‐Rodríguez et al., 2021). They use statistical and machine‐learning approaches to relate species' observations to environmental predictor variables and identify the main environmental determinants of species' ranges (Zurell & Engler, 2019). One of their purposes is to provide estimations of the species environmental preferences, allowing for extrapolation to other conditions in space or time, supporting the analysis of a wide variety of biodiversity patterns, and facilitating the understanding of both past and future distributions of species, which plays a crucial role in biogeographical studies.

Thus, in this study, we used records for *M. ruber* and *M. keaysi* to predict the ecological niche model of these species into the past and future, understanding how their distributions and areas of environmental suitability were shaped during historical periods of great environmental change. Our objective was to infer which environmental factors were important in shaping their areas of potential suitability and project them into the future to assess changes in the dynamics of areas environmentally suitable for the survival of species in climate change scenarios. From this, understanding how medium‐term climate change in the future can affect the potential distribution patterns of these groups of bats can help in efforts to protect these species.

## 2 METHODS

The occurrence records for *Myotis ruber* and *Myotis keaysi* were gathered by this research team based on the analyses of museum specimens deposited in the following collections (Appendix S1). Specimens were identified following criteria defined by Moratelli, Burgin, et al. (2019), totaling 36 occurrence localities recovered for both species, representing their entire distribution ranges (Table S1).

The climatic data used were obtained from the WorldClim 2.1 (https://www.worldclim.org, a public repository online) database, representing a set of 19 variables related to precipitation and temperature used to predict the current and future potential distribution of species of *Myotis*. In the same way, we also used the Paleoclim (http://www.paleoclim.org, a public repository online) database to obtain climate variables for past periods for projecting the potential distribution of *Myotis* species. For the past, we used the following time periods: Early Pleistocene (787 ka); Middle Pleistocene (last interglacial, 130 ka); and Late Pleistocene (last glacial maximum, 21 ka) because they represent periods of great environmental changes that occurred across the planet in the past (Griffing et al., 2022). For the future, we considered the short‐term (2061–2080) and medium‐term (2081–2100) periods. For all the projections, we used the bioclimatic variables at a 2.5 arcmin resolution (~4 km; Hijmans et al., 2005; Brown et al., 2018). For the model, overlapping occurrence points or those with less than 5 km distance between them were also not used. For future models, we used the Global Circulation Model MRI‐ESM2.0, which underwent numerous improvements meant for highly accurate climate reproducibility (Yukimoto et al., 2019). Shared socioeconomic pathways (SSP) emission scenarios are based on five narratives: SSP 1, SSP 2, SSP 3, SSP 4, and SSP 5, that describe alternative socio‐economic developments, including sustainable development, regional rivalry, inequality, fossil fuel‐driven development, and intermediate development, being translated into economic growth, population change, and urbanization, to consequently represent different mitigation and adaptation challenges (Riahi et al., 2017). The SSP 2 scenario describes a path in which social, economic, and technological trends do not change markedly from historical patterns, but development and income growth proceed unevenly, and the SSP 5 scenario is based on a high dependence on fossil fuels, which results in higher CO_2_ emissions, rapid technological progress, and human capital development (Riahi et al., 2017). The climate projections used from CMIP6 (Coupled Model Intercomparison Project Phase 6—set of global models used in climate change analyses by the Intergovernmental Panel on Climate Change/IPCC) data were SSP 2–4.5 and SSP 5–8.5, intermediate and advanced scenarios of global climate change, respectively, and have estimated warming ranges of 2.1°–3.5°C in the intermediate emissions scenario and 3.3°–5.7°C in the very high emissions scenario (Pörtner et al., 2022).

The ecological niche modeling tends to exhibit variable predictions when using different algorithms (Diniz‐Filho et al., 2009). Therefore, we used five algorithms to obtain more robust predictions: Maxent, Random Forest, SVM, BIOCLIM, and DOMAIN. Models were built using the packages *sdm Version 1.1‐8* (Naimi & Araújo, 2016) and *dismo Version 1.3‐14* (Hijmans et al., 2017) in the *R* environment (R Core Team, 2023). For the construction of the models, we ran 10 replicates for each algorithm and time periods per species, generating 800 models in total (10 replicates × 5 algorithms × 8 time periods × 2 species).

To reduce the impact on the prediction process and improve the accuracy of the model, we used Spearman's correlation for the choice of variables in order to choose the variables least correlated with each other, thus the variables with a correlation lower than 0.6 were used to build the models. The importance of the variables was calculated using the function *varImp* (Probst & Janitza, 2020) in the *sdm* package. The following variables were selected from the Spearman's correlation for *M. ruber*: annual mean temperature (BIO1), temperature seasonality (standard deviation ×100) (BIO4), precipitation seasonality (coefficient of variation) (BIO15), and precipitation of warmest quarter (BIO18). For *M. keaysi*, the variables were as follows: mean diurnal range (BIO2), mean temperature of warmest quarter (BIO10), precipitation of driest month (BIO14), and precipitation of wettest quarter (BIO16).

To evaluate the performance of the models, the occurrences were divided into two subsets: one with 70% of the occurrences as training data (data used to fit the model) and another with 30% as test data (data used to evaluate the model). Consensus models were created from an ensemble of the selected models to reduce the influence of errors and increase the reliability of the final model (Araújo & New, 2006). Here, the consensus model used for modeling ecological niche of *M. ruber* and *M. keaysi* is the weighted average AUC of the five listed algorithms using the *sdm* package. The models were evaluated by two criteria: area under the curve (AUC) and true skill statistic (TSS). AUC values higher than 0.7 indicate that the models have good performance (Dudík et al., 2004). TSS ranges between −1 and 1, with values higher than 0.4 indicating that the models have fair quality (Landis & Koch, 1977). Here, we consider AUC > 0.8 and TSS > 0.5 as thresholds to measure the accuracy of the models; results below these values will be excluded from the final ensemble. To quantify the estimated distribution area for the species in each time period, continuous and binary environmental suitability maps were generated, where the areas on the binary maps were measured based on software *ImageJ* (Schneider et al., 2012) to measure and compare how the size of these areas varied between periods in relation to the current environmental suitability area.

## 3 RESULTS

The resulting environmental suitability maps of *Myotis ruber* and *Myotis keaysi* showed that the potential current distribution of *M. ruber* is mostly associated with the Atlantic Forest regions, while *M. keaysi* is mostly related to highlands in the Andean regions (Figure 1). The AUC and TSS values were considered good for the models, with the average values for all periods being: *M. ruber*, AUC = 0.910 ± 0.050; TSS = 0.825 ± 0.094 and *M. keaysi*, AUC = 0.889 ± 0.044; TSS = 0.781 ± 0.083.

**FIGURE 2:**
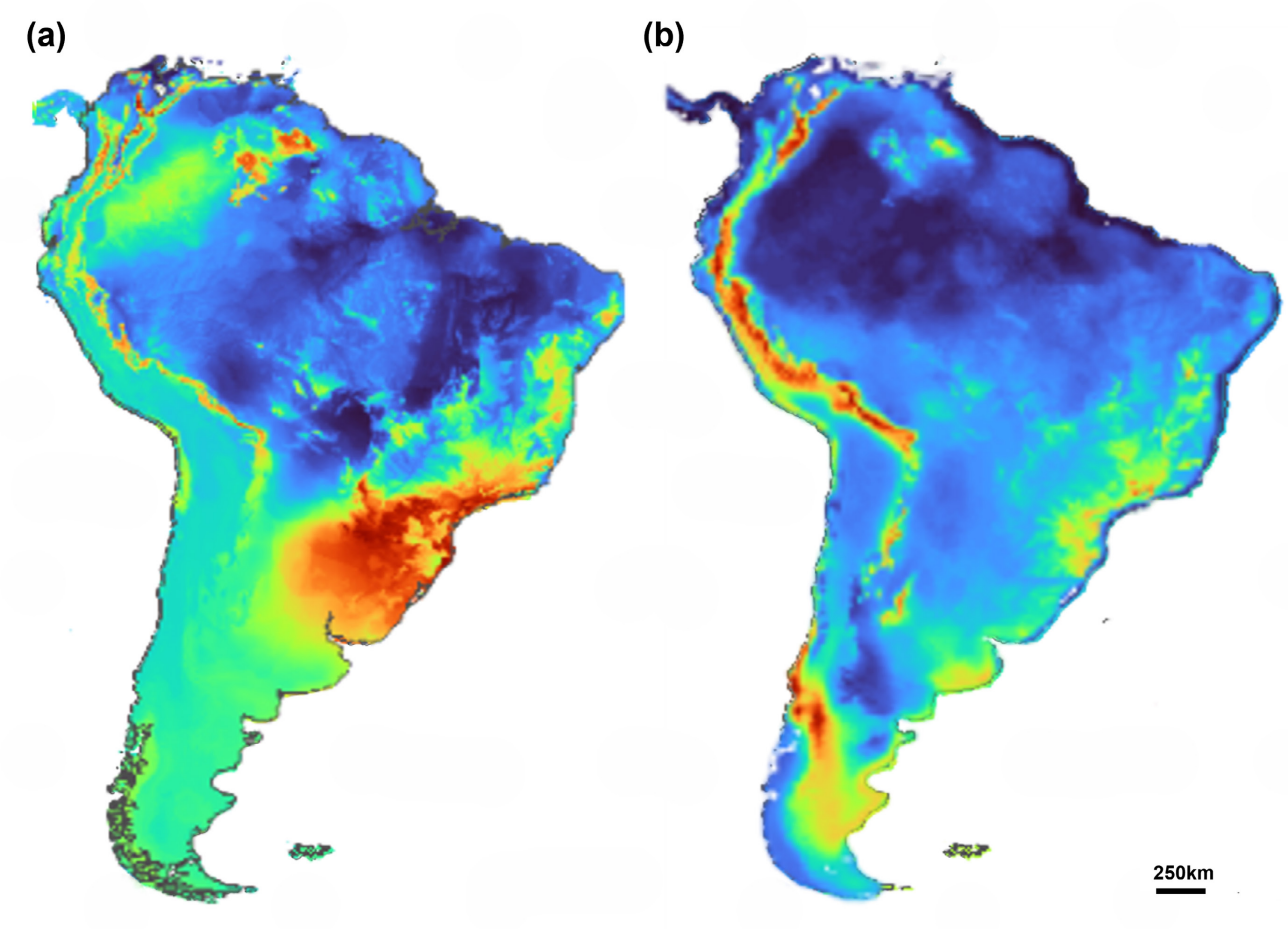
Current areas of potential environmental suitability for *Myotis ruber* (a) and *Myotis keaysi* (b). Warmer colors indicate greater suitability.

Currently, *M. ruber* and *M. keaysi* have opposite environmental preferences; *M. ruber* occurs in the Atlantic Forest regions from northeast Brazil to Northern Argentina, passing through regions of Paraguay; whereas *M. keaysi* has continuous distribution along the Andes, from southern Argentina to Venezuela, but its occurrence is not recorded for Colombia. The models generated confirm this current distribution and indicate areas that may currently be suitable for the survival of these species, but this does not mean that, despite being suitable, these areas will necessarily have occurrence of the species. The maps show that *M. ruber* also has small areas of environmental suitability that pass through the low‐altitude Andean regions going from northern Argentina to Venezuela (Figure 2a). Currently, the areas of environmental suitability for *M. ruber* are around 607,345.97 km^2^, which is equivalent to 3.4% of the total area of South America. *Myotis keaysi* occupies high‐altitude Andean areas, but the models generated show that there are suitable areas for the species further south in South America, in the regions of Chile and Argentine Patagonia (Figure 2b). The potentially suitable area of *M. keaysi* covers about 279,385.75 km^2^ of the current total area of South America, ca. 1.5% of the continent. For the current models, the climatic variable BIO1 (annual mean temperature, 52.5%) was more important for the projections of *Myotis ruber*, while for *Myotis keaysi*, the most important variable for the current projection was BIO10 (mean temperature of warmest quarter, 50.1%).

### 3.1 *Myotis ruber* potential suitability

#### 3.1.1 Potential suitability in the Early Pleistocene (787 ka)

**FIGURE 3:**
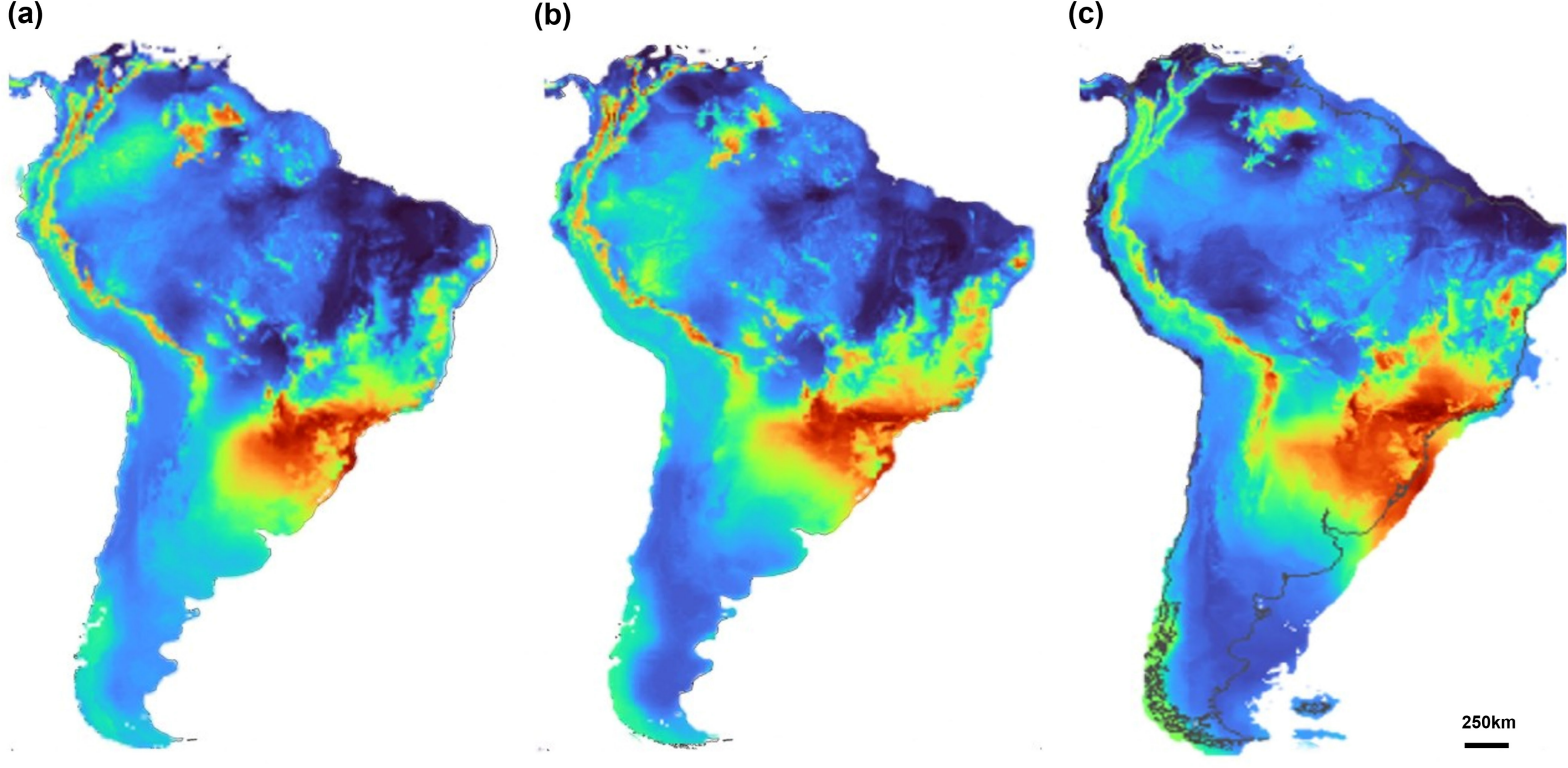
Areas of potential environmental suitability of *Myotis ruber* for the following periods in the past: 787 ka (a); 130 ka (b); 21 ka (c). Warmer colors indicate greater suitability.

Models for this period demonstrated satisfactory results with AUC and TSS values above the metrics used: 0.855 and 0.71, respectively. The models indicated that in addition to the areas in which it currently occurs, *M. ruber* would have areas of environmental suitability throughout the low‐altitude Andean regions, from Argentina to Venezuela. However, the southern regions of South America and central/northern Brazil do not present areas of environmental suitability for the species (Figure 3a). During this period, the variables that contributed most to the model's performance were as follows: BIO1 (annual mean temperature, 62.5%) and BIO15 (precipitation seasonality, 21.4%). In the Early Pleistocene, the estimated suitable area for the species in South America was about 13.9% smaller than the current potential area.

#### 3.1.2 Potential suitability in the Middle Pleistocene (130 ka)

During this period, the BIOCLIM algorithm had averages for AUC below the expected criteria (0.77). Despite this, the other algorithms used showed AUC and TSS values with an average of 0.9 and 0.78, respectively. The models maintain the suitable regions of the Early Pleistocene but also a moderate increase in suitable areas toward the northeast of Brazil and the beginning of a connection between the regions close to Paraguay and Bolivia (Figure 3b). The variables that most contributed to the model were BIO1 (annual mean temperature, 58.4%) and BIO4 (temperature seasonality, 19.6%). The total estimated suitable area for the species in South America in the Middle Pleistocene was about 11.9% larger than the current suitable area.

#### 3.1.3 Potential suitability in the Late Pleistocene (21 ka)

As in the Middle Pleistocene, the BIOCLIM algorithm presented AUC and TSS values below the average for the Late Pleistocene (0.73 and 0.47). Despite that, the other algorithms showed satisfactory AUC and TSS values, 0.93 and 0.86, respectively. During this period, we observed an increase in area of suitability with a concentration toward the coastal region of Brazil (Figure 3c). For this model, the most important variables were as follows: BIO1 (annual mean temperature, 35.2%) and BIO18 (precipitation of warmest quarter, 19.6%). In the Late Pleistocene, the total estimated suitable area for the species in South America was about 42.1% larger than the current suitable area, being the period of greatest potential for the distribution of the species during the Pleistocene.

#### 3.1.4 Potential suitability in the future (2061–2080/2081–2100 SSP 2–4.5)

**FIGURE 4:**
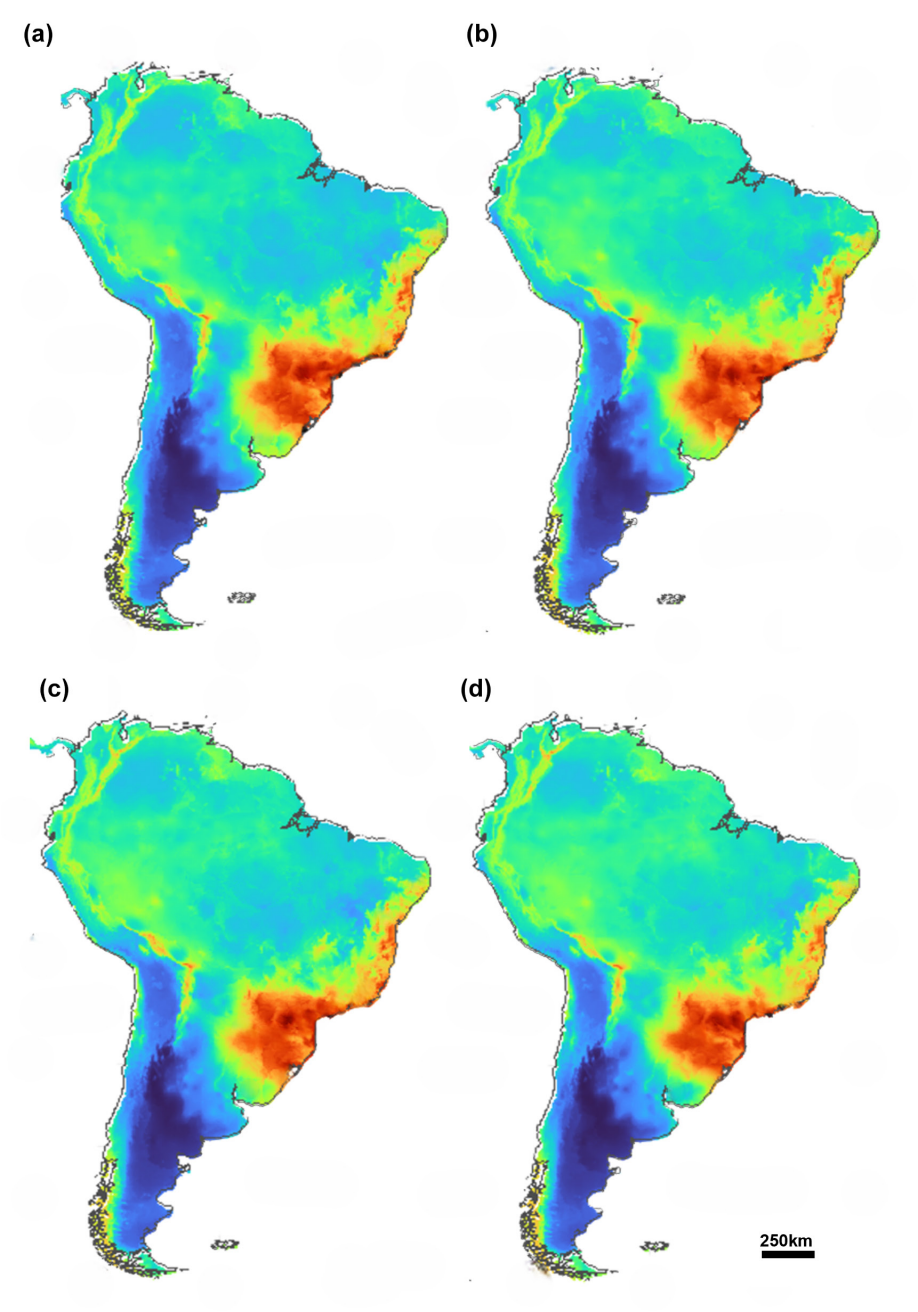
Areas of potential environmental suitability of *Myotis ruber* for the following periods in the future: 2061–2080/SSP 2–4.5 (a); 2081–2100/SSP 2–4.5 (b); 2061–2080/SSP 5–8.5 (c); 2081–2100/SSP 5–8.5 (d). Warmer colors indicate greater suitability.

For SSP 2–4.5 scenario of climate change, the algorithms showed AUC and TSS values of 0.914 and 0.828, respectively. 2061–2080 projections show a high decrease in suitability compared to current projections (Figure 4a). The main decrease in these environments affected the northern regions of South America, but the main difference occurred in the suitability regions located in Uruguay, Paraguay, Argentina, and southeastern Brazil. The variables that most contributed to 2061–2080 projections were BIO1 (annual mean temperature, 39.8%) and BIO4 (temperature seasonality, 27.8%). The projection for the intermediate scenario indicates that the suitable area will be 53.3% smaller than currently.

For the 2081–2100 projections, a small increase in suitable areas can be observed; mainly concentrated in the south and southeast regions of Brazil (Figure 4b). The ensemble AUC and TSS values were 0.85 and 0.782, respectively. In this model, the BIOCLIM algorithm had AUC and TSS results below the metric used (0.66 and 0.37). The variables that contributed the most were BIO1 (annual mean temperature, 40.4%) and BIO4 (temperature seasonality, 22.1%). The 2081–2100 projection for the SSP 2–4.5 scenario indicates that the potential distribution area will be 40.1% smaller than the current.

#### 3.1.5 Potential suitability in the future (2061–2080/2081–2100 SSP 5–8.5)

The 2061–2080 projections for SSP 5–8.5 scenarios show AUC and TSS values of 0.975 and 0.965, respectively. Projections in SSP 5–8.5 scenarios do not present significant differences in relation to projections in SSP 2–4.5 scenarios. In general, the south and southeast regions of Brazil continue to be the main suitability area for the species, being very similar to its current distribution (Figure 4c). BIO1 (annual mean temperature, 56.6%) and BIO4 (temperature seasonality, 13.7) contributed the most to the model. The 2061–2080 projection for the SSP 5–8.5 scenario indicates that the suitable area will be 42.4% smaller than current.

The 2081–2100 projections for SSP 5–8.5 scenarios do not present significant changes; they maintain the same suitability areas with a small increase in area for these projections. Most of these suitable areas range from the northeast to the south of Brazil (Figure 4d). The ensemble AUC and TSS values were 0.978 and 0.947, respectively. In this model, the BIOCLIM algorithm had AUC results below the threshold used (0.77). The variables that most contributed were BIO1 (annual mean temperature, 50.6%) and BIO4 (temperature seasonality, 17%). The 2081–2100 projection for the SSP 5–8.5 scenario indicates that the suitable area will be 47.1% smaller than the current.

### 3.2 *Myotis keaysi* potential suitability

#### 3.2.1 Potential suitability in the Early Pleistocene (787 ka)

**FIGURE 5:**
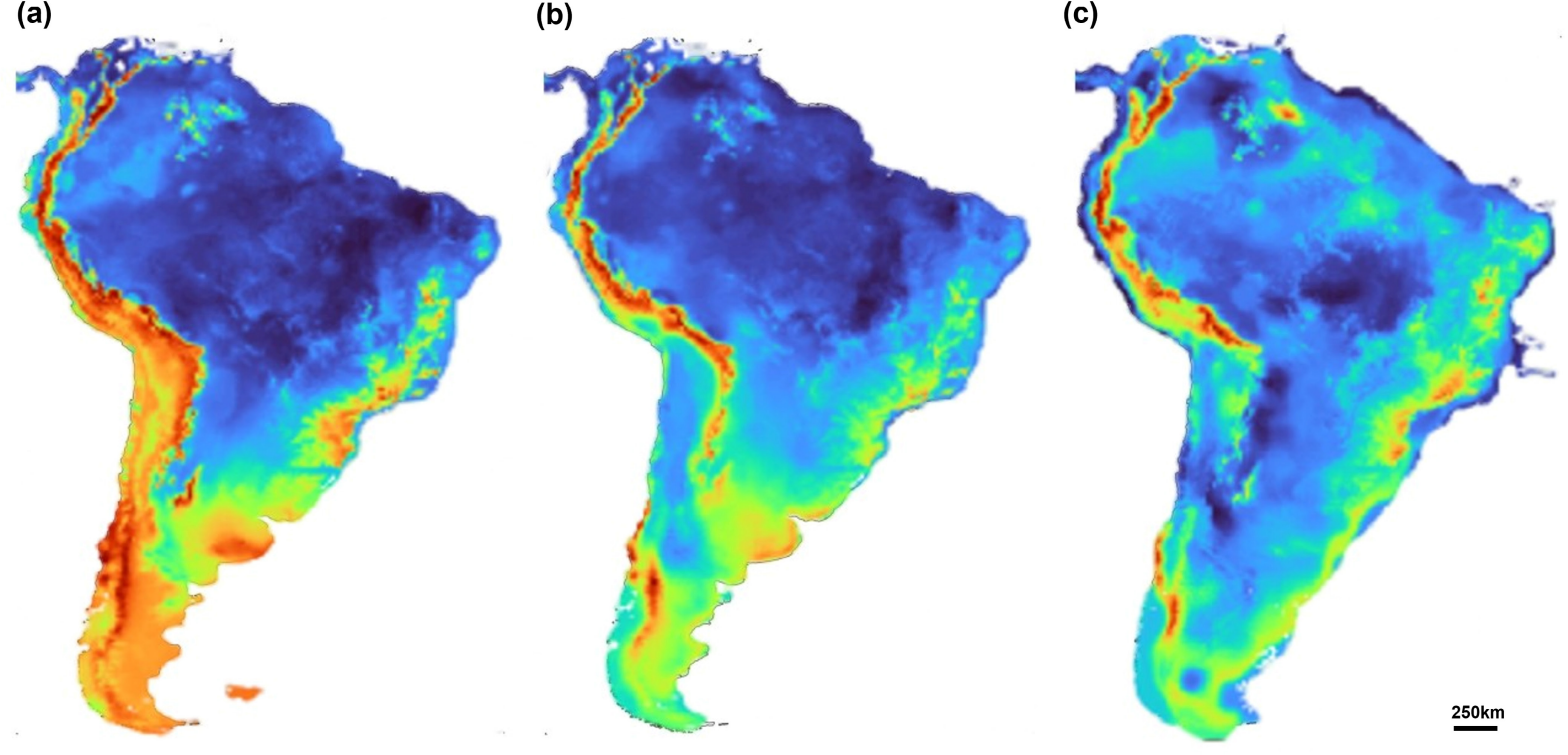
Areas of potential environmental suitability of *Myotis keaysi* for the following periods in the past: 787 ka (a); 130 ka (b); 21 ka (c). Warmer colors indicate greater suitability.

For this period, all algorithms showed satisfactory AUC and TSS values of 0.91 and 0.808, respectively. The suitable regions for *M. keaysi* are well marked and comprise the entire length of the Andes, from Venezuela to the extreme south of Chile and Argentina, including regions of Patagonia (Figure 5a). The model also indicates the presence of environmental suitability in regions of the northeast and southeast of Brazil. For this period, the most important variables were BIO10 (mean temperature of warmest quarter, 81.1%) and BIO16 (precipitation of wettest quarter, 9%). In the Early Pleistocene, the total estimated suitable area for the species in South America was 236% larger than the current period.

#### 3.2.2 Potential suitability in the Middle Pleistocene (130 ka)

In the Middle Pleistocene, the algorithms showed satisfactory AUC and TSS values of 0.86 and 0.694, respectively. During this period, the connections that existed between areas with the potential for the species to survive were broken, and it is possible to observe an intense decrease in areas suitable for the species, mainly in the extreme south of the continent (Figure 5b). The variables that most contributed to the model were BIO10 (mean temperature of warmest quarter, 72%) and BIO2 (mean diurnal range, 12.1%). In the Middle Pleistocene, the total estimated suitable area for the species in South America was about 103.3% larger than the current suitable area.

#### 3.2.3 Potential suitability in the Late Pleistocene (21 ka)

During this period, the average AUC and TSS values were 0.905 and 0.80, respectively, although BIOCLIM did not show satisfactory AUC values for this model (0.74). The Late Pleistocene continued to show a decrease in suitability for the species, which is visible mainly in southern Chile and along the Andes. During this period, suitable areas become even more concentrated and come closer to the suitability currently shown (Figure 5c). In Brazil, the northeast region still maintains few suitable areas, and a small new spot of potential area appears in the Amazon. The most important variables were BIO10 (mean temperature of warmest quarter, 50.8%) and BIO2 (mean diurnal range, 33.1%). In the Late Pleistocene, the total estimated suitable area for the species in South America was about 17.7% larger than the current suitable area.

#### 3.2.4 Potential suitability in the future (2061–2080/2081–2100 SSP 2–4.5)

**FIGURE 6:**
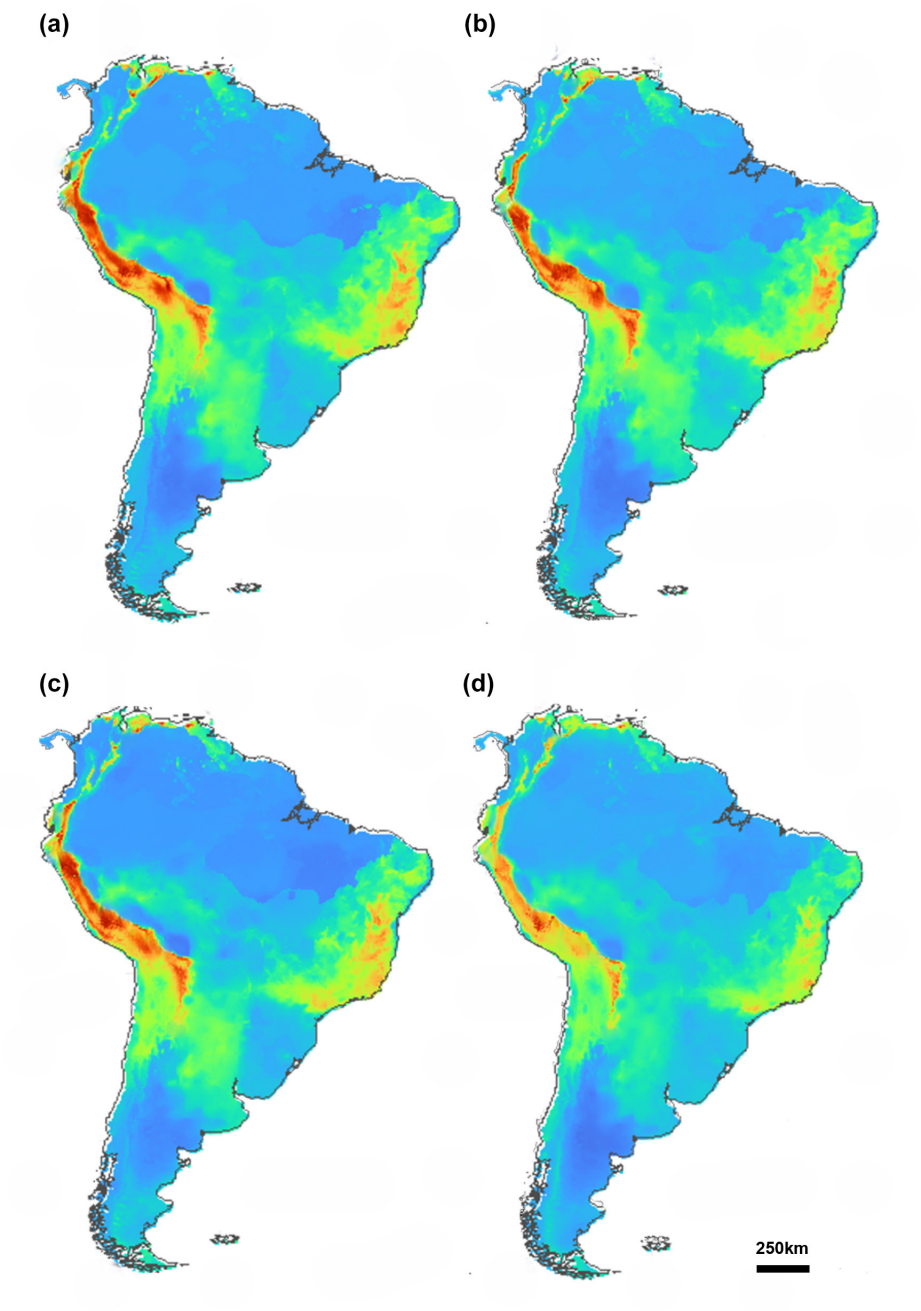
Areas of potential environmental suitability of *Myotis keaysi* for the following periods in the future: 2061–2080/SSP 2–4.5 (a); 2081–2100/SSP 2–4.5 (b); 2061–2080/SSP 5–8.5 (c); 2081–2100/SSP 5–8.5 (d). Warmer colors indicate greater suitability.

In the 2061–2080 projections in SSP 2–4.5 scenario, the algorithm BIOCLIM resulted in AUC and TSS values below the value used for projections (0.59 and 0.17), while the rest of the ensemble averaged to 0.84 and 0.70, respectively. The projections for this period indicate that the areas potentially suitable for the presence of the species follow the same design as the current and Late Pleistocene models. The Andean region continues to be the area with higher suitability. There were also patches of high suitability in the Bahia State of northeastern Brazil. However, the connection between these areas is practically nonexistent (Figure 6a). The most important variables for this model were BIO10 (mean temperature of warmest quarter, 30%) and BIO2 (mean diurnal range, 15%). The 2061–2080 projection for the SSP 2–4.5 scenario indicates that the suitable area will be 36.4% smaller than currently.

For the 2081–2100 projections, a minimal difference is generally observed (Figure 6b). The difference in projections is the apparent decrease of suitability patches in northeastern Brazil and a small decrease also in the areas of the Andean region. For this projection, the BIOCLIM algorithm also showed AUC and TSS values below acceptable limit (0.67 and 0.33). For the other algorithms, the AUC and TSS values were 0.97 and 0.92, respectively. BIO10 (mean temperature of warmest quarter, 33%) and BIO2 (mean diurnal range, 20.4%) were the most important variables for the species distribution. The 2081–2100 projection for the SSP 2–4.5 scenario indicates that the suitable area will be 44.8% smaller than the current.

#### 3.2.5 Potential suitability in the future (2061–2080/2081–2100 SSP 5–8.5)

The 2061–2080 projections in SSP 5–8.5 scenarios showed that again, the BIOCLIM algorithm was not a good model, with AUC and TSS values of 0.72 and 0.47, respectively. Despite this, the other algorithms averaged 0.907 (AUC) and 0.86 (TSS). These models almost entirely exclude the Amazon region and areas below 40° latitude as suitable areas (Figure 6c). The Andean region is the area with higher suitability, as well as in the SSP 2–4.5 scenario, and the northeast of Brazil continues to have small suitability areas. The 2061–2080 projection for the SSP 5–8.5 scenario indicates that the suitable area will be 44.1% smaller than current. The variables that shaped this model were BIO10 (mean temperature of warmest quarter, 64.9%) and BIO2 (mean diurnal range, 24%).

For 2081–2100 projections, the ensemble AUC and TSS values were 0.83 and 0.71, respectively. The BIOCLIM and DOMAIN algorithms showed the worst values of AUC (0.6 and 0.65) and TSS (0.27 and 0.47). These projections were the worst of all models (Figure 6d). A sharp decrease in suitability is observed in the northeast region of Brazil. The decrease in suitability in the Andean region is also visible, with few areas of high suitability. As well as for SSP 2–4.5 scenarios, the most important variables were BIO10 (mean temperature of warmest quarter, 51.8%) and BIO2 (mean diurnal range, 29.2%). The 2081–2100 projection for the SSP 5–8.5 scenario indicates that the suitable area will be 66.8% smaller than the current.

## 4 DISCUSSION

**FIGURE 7:**
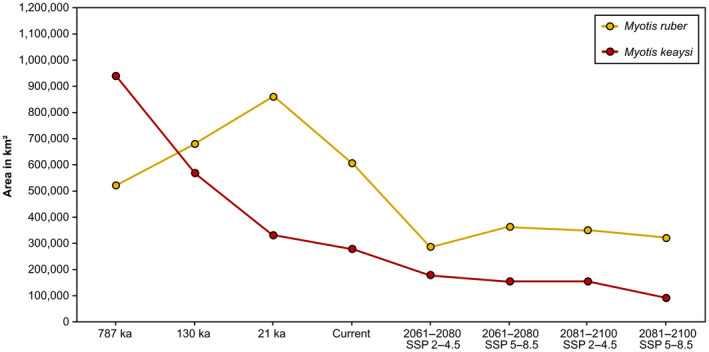
Changes in the potential distribution area (in km^2^) of *Myotis ruber* and *Myotis keaysi* in different periods.

Changes in the potential distribution of these species were analyzed by comparing ecological niche model results among current, past, and future climate scenarios. For both species, models for past scenarios predicted more areas with high environmental suitability. The greatest habitat losses (in km^2^) from one period to another were observed during the Pleistocene for *M. keaysi*. Despite this, these losses did not reach the levels observed in future models (Figure 7). We observed that environmental changes in the Pleistocene affected the suitable areas for species in different ways. This is because while *M. keaysi* lost suitability over time, *M. ruber* gained. However, the scenario changes in relation to future models where both species only lose suitable areas.

### 4.1 Historical biogeography and evolutionary issues

In general, both species have the potential to occur in South America at latitudes ranging from 10° N to 35° S. Regions below 39° S were not favorable to *M. ruber*, which is interesting as this latitude is home to the structure of Huincul High in Neuquén Basin, Argentina. This region is the northern limit of the Patagonia region and consists of an intraplate deformation belt perpendicular to the Pacific convergent margin. It was developed along the Permian suture of the Patagonia terrane with Western Gondwana (Ramos, 1984; Suárez et al., 2019), which may have acted as a kind of environmental barrier that seems to have had some influence on the distribution of *M. ruber*. Studies focused on South American megafauna, for example, have already reported the nonoccurrence of certain species below this latitude (Araújo et al., 2021).

During the Early Pleistocene, the continent was under the influence of glacial events such as the Great Patagonian Glaciation (GPG; Griffing et al., 2022). Therefore, it can be inferred that the regions suitable for the existence of both species had lower temperatures than today. For *Myotis keaysi*, this was the period of greatest environmental suitability, which may indicate, due to the location of these areas of suitability, that *M. keaysi* occurred in very cold and arid environments. The study of van der Hammen (1974), based on palynological data for the Andean region in this period, indicates that there were several conspicuous recurrent changes in the vegetation cover from Andean Forest to open Paramo (and vice versa), resulting in a downward and upward displacement of vegetation zones with the climate colder than it is today at the same elevation. The environments suitable for the survival of *M. keaysi* during this period demonstrate that the species preferred areas with lower average temperatures. The species probably managed to adapt from open landscapes to landscapes with greater vegetation cover since in that period this was the formation of the vegetation in these regions (van der Hammen, 1974). During this period, we also observed connection points between potential areas for *M. keaysi* and *M. ruber*.

On the other hand, for *Myotis ruber*, Early Pleistocene could have been the least suitable period for the species in the past, leading to zero probability of occurrence in high‐altitude areas in the Andes and southern South America. The maps show that the main areas of suitability for the species are concentrated and inserted in the Atlantic Forest, region that runs from Uruguay to southeastern Brazil, passing through regions in Argentina and Paraguay. In the Pleistocene sequences of Argentina, loess and paleosols also show fluctuations between glacial and interglacial cycles that occurred during the Pleistocene (Soibelzon & Tonni, 2009). In the region where the Atlantic Forest is located, the amount of precipitation and low temperatures of the early Pleistocene were greatly influenced by the southern latitude circulation. These climate fluctuations showed a different dynamism with an increase in cool forests and drops in relative temperature, concomitant with regional long‐trend cooling observed in marine records (Rodríguez‐Zorro et al., 2022). The areas suitable for *M. ruber* demonstrate that despite the lower temperatures in this period, the species was also influenced by variability in precipitation and preferred more humid regions with denser vegetation.

In the Middle Pleistocene, the projections show that the increase in temperature positively influenced the distribution of *M. ruber* and decreased suitability for *M. keaysi*. This period is recognized as the Last Interglacial (LIG), which would be the most recent geological period during which conditions were similar to the present interglacial but with small anthropogenic effects and the warmest time period of the last 200 ka (Otto‐Bliesner et al., 2013). Clearly, the increase in precipitation and temperature acted in opposite ways on the distribution of the two species. In this sense, van der Hammen (1974) indicates that the gradual enrichment of the flora in this interglacial period resulted in the development of Paramo vegetation and Andean Forest on the mountain slopes surrounding the high plain. Paleoclimatic interpretations for the Atlantic Forest region indicate an extratropical regime with rainfall occurring mainly in winter at that time. The presence of the tree Araucaria and a dense rainforest during the interglacial provides evidence for the occurrence of both winter and summer precipitation regimes in the area (Ledru et al., 2009). Furthermore, sedimentological studies indicate that the sea level in that period was 7 m above the current level (Tomazelli & Dillenburg, 2007). For regions in Argentina close to Paraguay and Uruguay, humid and hot conditions are recognized without any taxonomic indicator of cold‐dry environments (Pardiñas et al., 2004).

Inferring from this, it appears that the increase in precipitation, which also seems to have favored environments with dense vegetation in these regions suitable for *M. ruber*, mainly enabled the presence of an environment more conducive to the existence of *M. ruber*. As for *M. keaysi*, the increase in temperature seems to have been the decisive factor in the reduction and concentration of areas suitable for the species, which apparently caused the species to concentrate in high‐altitude regions where temperature and humidity were higher. Despite the clear difference in their environmental preferences, this was a period where connections between species' suitable ranges were closer. For *M. ruber* that had an increase in suitability enabling this connection, this may mean greater ecological plasticity even if certain characteristics of the environment are more favorable to *M. ruber* than to *M. keaysi*.

The Late Pleistocene was recognized as the Last Glacial Maximum (LGM), was a major climatic event in the Last Glacial Cycle (LGC), and in many areas, was a time of significant landscape change (Hughes, 2021; Ray & Adams, 2001). In this period, the greatest mass of ice was present on Earth, showing up in ice cores and carbonates as a peak of ^18^O (Crowley & North, 1991). Due to the extent of the ice sheets during LGM, coastlines were dramatically changed in certain areas, and based on coral cores, a commonly accepted mean value for this drop is about 120 m (Ray & Adams, 2001). Contrary to projections for previous periods, the LGM was less favorable to the potential distribution of *M. keaysi* than *M. ruber*. Since a colder and drier climate than today was expected, in addition to a significant reduction in forest areas, giving place to deserts and semi‐desert areas would be a more favorable environment for *M. keaysi*. However, pollen and diatom records combined with carbon and nitrogen isotopes suggest instead that cool, humid forests persisted, with no evidence of forest retraction throughout the LGM and the Atlantic Forest, for example, and would not have been restricted to small patches because forest cover would have extended onto the continental shelf even if its composition is different from the current one (Leite et al., 2016). In the Amazon region, savannah expanded through the southeast margins of Amazonia to connect with the nonforested biomes in the far north, while moist forest remained robust along the Atlantic coast in northern Brazil, showing no indication of a trans‐Amazonian Atlantic corridor at the biome level, although canopy density and height were slightly reduced (Sato et al., 2021). Isotopic studies also indicated that the Amazon region was drier during the glacial period, but despite this, the tropical forest persisted throughout this time (Wang et al., 2017). Thus, the conservation of certain types of environments preferred by *M. ruber*, despite the period, may have helped to increase suitability for the species. In addition, the decrease in temperature in these regions seems to have reached tolerable levels for the species, since temperature variables were the most important for the model. In the Tropical Andes region, which showed the highest concentration of suitability for *M. keaysi* in this period, evidence suggests that cold events in the northern hemisphere were accompanied by increases in precipitation in these regions, caused by large and rapid changes in the hydrological balance in the tropics (Fritz et al., 2010). Icefields in the Southern Andes were most expansive when global temperatures and sea levels were lowest because Pacific westerlies supplied abundant moisture. Furthermore, the increase in effective moisture at this time was probably linked to atmospheric cooling (Clapperton, 1993). Although the current distribution of *M. ruber* and *M. keaysi* is shaped by different environmental factors, past projections helped to understand that, possibly despite different environmental preferences, *M. ruber* would be much more flexible than *M. keaysi* to the changes that were taking place in the environment, especially in terms of temperature. By correlating the environmental changes that the continent was going through and the potential distribution of these species, since temperature and humidity were important variables for *M. keaysi*, it is possible to infer that this species is more sensitive to changes in temperature and that the substantial reduction in vegetation cover in these regions may not have been the main factor that influenced its environmental suitability. And *M. ruber*, despite adapting well to changes in temperature, is also influenced by changes in precipitation and prefers areas of dense vegetation.

*Myotis ruber* and *Myotis keaysi* are phylogenetically close, and some phylogenies based on mitochondrial genes have identified them as sister species (e.g., Larsen et al., 2012; Novaes et al., 2021, 2022). However, the phylogenetic relationships of these species with the rest of the ruber group are still not fully understood, as studies using different genes have found distinct topologies (Carrión‐Bonilla & Cook, 2020; Stadelmann et al., 2007). The diversification of the ruber group occurred during the Plio‐Pleistocene and estimates of the genetic divergence between *M. ruber* and *M. keaysi* are greater than 10%, a considerably high distance for species of Neotropical *Myotis* (Novaes et al., 2021; Stadelmann et al., 2007). Our past distribution models suggest the existence of highly suitable areas for secondary contact between *M. ruber* and *M. keaysi* in the Middle Pleistocene (130 ka). These regions in Paraguay and northern Argentina may have favored hybrid zones with the possibility of gene flow, generating trans‐species polymorphism (Schad et al., 2012), which explains the low resolutions in the phylogenies (i.e., Carrión‐Bonilla & Cook, 2020; Novaes et al., 2021; Stadelmann et al., 2007). If the phylogenetic hypothesis recovers *M. ruber* and *M. keaysi* as sister species is confirmed, the ENM results indicate an allopatric speciation with partial ecological niche conservatism, which reflects the phenotypic similarity.

### 4.2 Future projections, areas of potential climate stability, and species conservation

Contrary to past inferences, projections for the future of species are much more pessimistic for both scenarios. The projections for *M. ruber* concentrate its areas of environmental suitability in the future in both scenarios, in areas from the Atlantic Forest to the northeast region of Brazil, losing almost completely its suitability in the Andean regions. According to the literature, these regions of reduced suitability present an increase in temperature and aridity, which would affect the Amazon region on a large scale around 2070 (Cavalcante et al., 2020). Sales et al. (2015) indicate for the northeast region of Brazil an increase of about 2.8°C in temperature and precipitation. For the most southern region of Brazil, air temperatures are projected to rise by an average of 2.5–3.5°C (Marengo et al., 2011). In past periods, the potential areas for *M. ruber* were areas that presented a good volume of precipitation, so the decrease in suitability for the species can be seriously affected by an increase in temperature and an increase in aridity in South America. Despite that, Scarano and Ceotto (2015) believe in the possibility of the Atlantic Forest no longer being a point of decline in biodiversity but rather a point of climate adaptation due to the high ecological plasticity and adaptive capacity of many species in the Atlantic Forest. This information can be considered when we analyze the future distribution (it can also be observed in models for the past) of *M. ruber*, which has a large part of its potential concentrated in areas identified as belonging to the Atlantic Forest.

Like *M. ruber*, *M. keaysi* shows a tendency to decrease and concentrate its potential distribution in future projections. In the case of *M. keaysi*, the central Andes became practically the only suitable area for the species. Both sides of the Andes exhibit warming year‐round, but particularly east of the Andes over Amazonia and the South American monsoon region, according to Marengo et al. (2011). Despite important losses being projected for several biomes, projections suggest that between 74.8% and 83.1% of the current total tropical Andes will remain stable, depending on the emission scenario and time horizon. However, several biomes are projected to lose more than 30% of their current area. Vulnerable areas and the biomes that are currently most threatened (glaciers and cryoturbated areas, paramo, and evergreen montane forest) but also specific areas under stress due to changes in physiognomy or humidity levels (Tovar et al., 2013). According to Marengo et al. (2011), in all future climate scenarios, South America will experience significant changes in precipitation and temperature extremes, including in the northeastern region of Brazil, an increase in the frequency of dry days. Anjos et al. (2021), in their specific research for the SSP 5–8.5 scenario, confirm that the terrestrial biomes of South America would be exposed to climatic conditions that are not analogous to the current ones, with a trend of annual temperature increase and a significant reduction of humidity in the forest biomes. The concentration of the distribution of *M. keaysi* in the future projections for both scenarios can infer that, as in the past, the species is more sensitive to changes in the environment than *M. ruber*, and in this case, the increase in temperature seems to be the main factor shaping the future distribution of the species.

**FIGURE 8:**
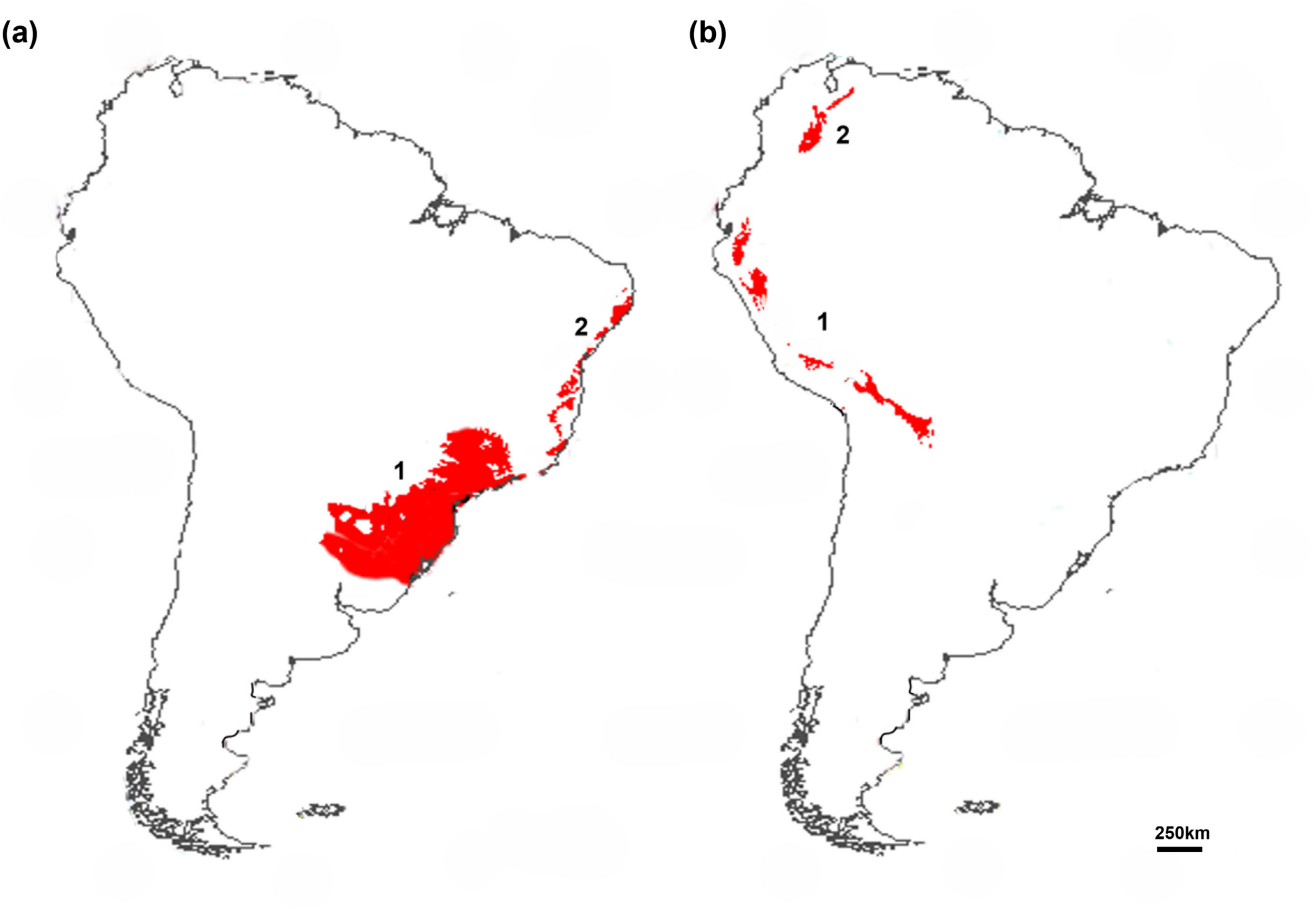
Areas of potential climate stability for *Myotis ruber* (a) and *Myotis keaysi* (b), with high suitability for the species occurrence in both past and future projections.

In the past, climatically stable areas were frequently associated with high levels of diversity and endemism, and in the future, these areas may perform a similar role in protecting current biodiversity. Thus, they are considered to be very important buffers against the impacts of rising global temperatures and wide climate changes (Werneck et al., 2012). In this work, areas of potential climate stability were inferred for the two species of *Myotis*, and the criteria used initially were the choice of areas that have a high potential for the species to occur since the Early Pleistocene (Figure 8). For each species, two main areas of stability were identified based on that criterion: for *M. ruber*, the first region is present on the Brazilian coast from the southeast to the south of the country, and extending inland and reaching regions of Uruguay, Paraguay, and Argentina. The second region extends along the coast of northeastern Brazil (Figure 8a). Most of these areas are located in the Atlantic Forest biome, which currently has high average temperatures and air humidity throughout the year and regular and well‐distributed rainfall, which allows a high plant biodiversity formed by forests of different types (Pereira, 2009). For *M. keaysi*, the first area of potential climate stability was identified in the Tropical Andes region, extending from southern Bolivia to Ecuador almost continuously, and the second area is in Venezuela (Figure 8b). These areas have very heterogeneous vegetation and climate and are among the most biodiverse regions on the planet (Myers et al., 2000).

Dinerstein et al. (2017) present data that indicate the protection status of the world's ecoregions. When we compare these stable areas for *M. ruber* and *M. keaysi* with the protection status of these ecoregions, most of these areas are in regions with “Nature Could Recover” and “Nature Imperiled” statuses. These statuses indicate that most environments have enough undisturbed habitat to recover, but some have an average of only 4% natural habitat remaining. Our data indicate that these areas of potential climate stability may function as refuges for animal and plant species. Care needs to be taken when referring to climatically stable areas because this relationship also depends largely on species‐specific aspects. Areas that are climatically stable over time may have this stability reflected in ecological and evolutionary patterns, such as abundance and morphology. In this way, our study can be used as a starting point for important studies to be developed in the future in support of conservation strategies for habitats that could be a refuge for a great diversity of species in a future that is not so optimistic for environmental preservation.

## 5 CONCLUSION

According to our findings, the areas of climatic suitability of *Myotis ruber* and *Myotis keaysi* are mainly influenced by changes in temperature, and, in addition, for *M. ruber*, the precipitation regime also becomes important. Furthermore, *M. ruber* appears to have been more flexible to decreases in temperature that occurred in the past, which allowed it to expand its areas of environmental suitability, unlike *M. keaysi*, which decreased and concentrated these areas. In future scenarios of drastic increases in temperature and decreases in humidity, both species are projected to show a range loss, and the flexibility observed in the past will no longer be observed. Despite this, *M. ruber* will in the future have larger areas of potential suitability than *M. keaysi*. The inference of areas of potential climate stability is an important step in efforts to conserve and preserve species, but it needs to be done carefully, considering other aspects that may influence this stability.

## AUTHOR CONTRIBUTIONS

**Karoliny de Oliveira:** Conceptualization (equal); methodology (equal); project administration (equal); resources (equal); visualization (equal); writing – original draft (equal); writing – review and editing (equal). **Roberto Leonan M. Novaes:** Data curation (lead); supervision (equal); writing – review and editing (equal). **Marcelo M. Weber:** Supervision (equal); writing – review and editing (equal). **Ricardo Moratelli:** Conceptualization (equal); supervision (equal); writing – review and editing (equal).

## FUNDING INFORMATION

This work was supported by Fundação Carlos Chagas Filho de Amparo à Pesquisa do Estado do Rio de Janeiro, Brazil (FAPERJ; KO‐E‐22/2021D2; RM‐E‐26/200.967/2021; RLMN‐E‐26/204.243/2021; E26/200.631/2022; and E26/200.395/2022), and Conselho Nacional de Desenvolvimento Científico e Tecnológico, Brazil (CNPq; RM‐313963/2018‐5).

## CONFLICT OF INTEREST STATEMENT

The authors declare no conflict of interest.

## Supporting information


Appendix S1.

Table S1.

## Data Availability

The authors confirm that the data supporting the results of this study are available in the Supplementary Material of this article or can be accessed on GitHub at: https://abre.ai/i9zW. All climatic data were downloaded from www.worldclim.org and www.paleoclim.org.
