# Supplementary material for: Forecasting climate change impacts on neotropical Myotis: Insights from ecological niche models for conservation strategies
Source: Ecol Evol. 2024 Jun 25;14(6):e11419. doi: 10.1002/ece3.11419 (PMC11199191; doi:10.1002/ece3.11419)
Supplement: Supplementary file 1 — Appendix S1. [file ECE3-14-e11419-s001.docx]

**Appendix S1.** List of specimens of *Myotis ruber* and *Myotis keaysi* used in the analyses. Voucher material consists of stuffed skins, fluid-preserved specimens, and skulls, deposited in the following museum collections: Coleção Adriano Lucio Peracchi at the Universidade Federal Rural do Rio de Janeiro (ALP, Seropédica, Brazil), Museu Nacional da Universidade Federal do Rio de Janeiro (MN and JAO, Rio de Janeiro, Brazil), Museu de Zoologia da Universidade de São Paulo (MZUSP, São Paulo, Brazil), Coleção de Mamíferos da Universidade Federal de Pernambuco (UFPE, Recife, Brazil), Colección Mamíferos Lillo at the Universidad Nacional de Tucumán (CML, Tucumán, Argentina), Museo Argentino de Ciencias Naturales "Bernardino Rivadavia (MACN, Buenos Aires, Argentina), Carnegie Museum (CM, Pittsburgh, USA), Museum of Texas Tech University (TTU, Lubbock, USA), American Museum of Natural History (AMNH, New York, USA), Museum of Vertebrate Zoology of the University of California (MVZ, Berkeley, USA), Field Museum of Natural History (FMNH, Chicago, USA), Museum of Zoology of the Louisiana State University, (LSUMZ, Baton Rouge, USA), Smithsonian National Museum of Natural History (USNM, Washington D.C., USA), Sam Noble Oklahoma Museum of Natural History (OMNH, Norman, USA), Royal Ontario Museum (ROM, Toronto, Canada), Muséum National d'Histoire Naturelle (MNHN-Paris, France), Muséum d'Histoire Naturelle de la Ville de Genève (MHNG, Genebra, Switzerland).

*Myotis keaysi* (N = 43): Argentina: Tucumán, Burruyacú (CML 6177, CML 7600, CML 8938, CML 9839, MACN 16795, MACN 16855, MACN 16857, OMNH 23499, 36207, TTU 32588). Bolivia: Cochabamba, Incachaca (CM 5256); La Paz (TTU 23951). Ecuador: Tungurahua, Pillaro, San António (AMNH 67607). Peru: Puno, Inca Mines (AMNH 15814 [holotype]), Ocaneque (MVZ 116050); Cuzco, Cordillera Vilcabamba.

*Myotis ruber* (N = 59): Argentina: Formosa, Laguna Blanca, Parque Nacional Pilcomayo (CML 4666, 4673, 4676, 4686); Misiones, Guarani, jat. Hwy 21, Arroyo Oveja Negra, ~2 km W Parque Provincial Moconá (CML 3877); Misiones, Parque Nacional Iguazú, Ayui (MACN 18490); Misiones, Loreto (MACN 18035); Misiones, Parque Nacional Iguazú, Seccional Mbocay (OMNH 18882). Brazil: Pernambuco, Brejo da Madre de Deus (UFPE 1022, 1026, 1089; ), Caruaru (UFPE 1105, 1285, 1361); Bahia, Alagoas (MHNG 1884-50); Minas Gerais, Viçosa (USNM 391140, ROM 70911, 78803–78805, 91211); Rio de Janeiro, Teresópolis (ALP 6452, 6457, 6458, 6497, 6499, 6506, 6512, MN 3400), Reserva Biológica do Tinguá (ALP 6621, 6683), Macaé de Cima (JAO 1751, 1756, 1773); São Paulo, Cananéia (MZUSP 27595), Boracéia (MZUSP 28359, 28367, 28368), São Paulo (MZUSP 31470–31473), Buri (MZUSP 32971–32973, 32975), Salesópolis (MVZ 185692); Santa Catarina, Nova Teotonia (MHNG 1916-71); Rio Grande do Sul, São Lourenço da Mata (MZUSP 1988). Paraguay: Sapucay, Sapucay (USNM 115097 [holotype], 121478); Itapua, Arroyo Pirayu (MHNG 1747-56), Santo Temembey, 4 km amont (MHNG 1695-27); Uruguay: Arroyo Grande (MHNG 1748-47).
