# Supplementary material for: Forecasting climate change impacts on neotropical Myotis: Insights from ecological niche models for conservation strategies
Source: Ecol Evol. 2024 Jun 25;14(6):e11419. doi: 10.1002/ece3.11419 (PMC11199191; doi:10.1002/ece3.11419)
Supplement: Supplementary file 2 — Table S1. [file ECE3-14-e11419-s002.pdf]

Table 1. Localities used to generate de ecological niche models of *Myotis ruber* and *Myotis keaysi*.

Species	Longitude	Latitude	Locality
M. ruber	-36.3775	-8.15347	Brejo da Madre de Deus, Pernambuco, Brazil
M. ruber	-35.96757	-8.34295	Caruaru, Pernambuco, Brazil
M. ruber	-40.30755	-12.43265	Alagoas, Sergipe, Brazil
M. ruber	-42.87399	-20.77804	Viçosa, Minas Gerais, Brazil
M. ruber	-43.00308	-22.45470	Teresópolis, Rio de Janeiro, Brazil
M. ruber	-43.45981	-22.59871	Tinguá, Rio de Janeiro, Brazil
M. ruber	-42.34654	-22.34427	Macaé de Cima, Rio de Janeiro, Brazil
M. ruber	-47.95735	-25.03404	Cananéia, São Paulo, Brazil
M. ruber	-45.89692	-23.74760	Boracéia, São Paulo, Brazil
M. ruber	-48.03312	-23.79125	São Miguel Arcanjo, Brazil
M. ruber	-52.39932	-27.21173	Nova Teutônia, Santa Catarina, Brazil
M. ruber	-52.03960	-31.45669	São Lourenço da Mata, Rio Grande do Sul, Brazil
M. ruber	-56.76060	-32.80832	Arroyo Grande, Uruguay
M. ruber	-56.96008	-25.67940	Sapucay, Sapucay, Paraguay
M. ruber	-54.99052	-26.72192	Arroyo Pirayú, Itapúa, Paraguay
M. ruber	-54.47699	-25.67906	Iguazú, Misiones, Argentina
M. ruber	-47.1004	-23.96657	Juquitiba, São Paulo, Brazil
M. ruber	-50.59972	-24.36837	Telêmaco Borba, Paraná, Brazil
M. ruber	-48.99424	-25.92733	Serra do Araçatuba, Paraná, Brazil
M. keaysi	-79.60308	-5.38065	Canchaque, Piura, Peru
M. keaysi	-79.50504	-5.96887	Olmos, Lambayeque, Peru
M. keaysi	-78.41885	-5.55834	La Peca, Amazonas, Peru
M. keaysi	-73.28989	-12.37670	Cordillera Vilcabamba, Cuzco, Peru
M. keaysi	-70.71753	-13.40240	Hacienda Cadena, Cuzco, Peru
M. keaysi	-76.08210	-9.71385	Cordillera Corphish, Huánuco, Peru
M. keaysi	-76.61260	-9.39462	Carpish 2, Huánuco, Peru

M. keaysi	-75.45736	-10.74538	Santa Cruz, Pasco, Peru
M. keaysi	-73.94456	-12.85384	Puncu, Ayacucho, Peru
M. keaysi	-70.04842	-15.81372	Limbani, Puno, Peru
M. keaysi	-75.39325	-11.10739	Chanchamayo, Junín, Peru
M. keaysi	-66.49800	-17.25825	Incachaca, Cochabamba, Bolivia
M. keaysi	-68.12127	-16.42049	La Paz, Bolivia
M. keaysi	-78.56438	-1.18566	Pillaro, Tungurahua, Ecuador
M. keaysi	-71.12286	08.57193	Montes de Milla, Mérida, Venezuela
M. keaysi	-64.8601	-26.44952	Burruyacú, Tucumán, Argentina
M. keaysi	-65.29250	-26.73159	Tafí Viejo, Tucumán, Argentina
